# The Science Underlying Giant Panda Conservation Translocations

**DOI:** 10.3390/ani13213332

**Published:** 2023-10-26

**Authors:** Yue Wang, Wei Wei, Feiyun Yuan, Dandan Cao, Zejun Zhang

**Affiliations:** 1 Key Laboratory of Southwest China Wildlife Resources Conservation (Ministry of Education), China West Normal University, Nanchong 637001, China; wangyue@stu.cwnu.edu.cn (Y.W.); weiw@cwnu.edu.cn (W.W.); cxy9@stu.cwnu.edu.cn (D.C.); 2 Liziping Giant Panda’s Ecology and Conservation Observation and Research Station of Sichuan Province, China West Normal University, Nanchong 637001, China; 3 Sichuan Lushi Expressway Co., Ltd., Chengdu 610041, China; chenyx@stu.cwnu.edu.cn; 4 Chengdu Normal University, Chengdu 611130, China

**Keywords:** artificial intervention, conservation translocation, giant panda, local population, self-sustaining

## Abstract

**Simple Summary:**

Pandas are a flagship species for global animal conservation and wild individuals are scarce. They are now segregated into 33 local populations, and 25 of them are too small to be self-sustaining. For these small populations, in addition to preservation of pandas in situ, translocation is one of the options for the species’ recovery. This paper reviews the scientific progress in conservation translocation of pandas, with the aim of providing theoretical guidance to improve the success rate of released pandas, and uses pandas as a model species to provide reference for the global conservation translocation of rare and endangered species.

**Abstract:**

The giant panda (*Ailuropoda melanoleuca*) is the flagship species of animal conservation worldwide, and the number of captive pandas reached 673 in 2021. According to the Fourth National Survey Report on the Giant Panda, there are 1864 wild pandas, segregated into 33 local populations, and 25 of these populations are too small to be self-sustaining. In addition to the conservation and restoration of panda habitats, conservation translocations, an approach that has been shown to be effective in slowing or reversing biodiversity loss, are highly desirable for panda conservation. The captive-bred panda population has grown rapidly, laying the foundation for releasing captive-bred pandas into the wild. This paper reviews the scientific advances in conservation translocations of pandas. Studies have shown that before translocation conservation programs are implemented, we should determine what factors are causing the depletion of the original population at the release site. The selection of suitable release sites and individuals will help to improve the survival rate of released individuals in the wild. Pre-release training and post-release monitoring are essential to ensure successful releases. We also see the great potential for increasing applications of Adaptive Management to improve the success of giant panda conservation translocation programs. This review provides theoretical guidance for improvement of the success rate in conservation translocations for captive pandas, and uses the panda as a model species to provide a global reference for the conservation translocations of rare and endangered species.

## 1. Introduction

The Earth is experiencing its sixth mass extinction, which, unlike the previous five, is largely human induced [1]. Biodiversity conservation has implications for the sustainable development of human societies and has become one of the most widespread concerns of the international community [2]. China is one of the world’s richest countries in terms of biodiversity and also one of the most threatened [3].

The giant panda (*Ailuropoda melanoleuca*) is undoubtedly the most discussed and represented species in China’s biodiversity conservation efforts. It is known as China’s national treasure because of its lovely appearance and the scarcity of individuals in the wild. China has made countless efforts to protect the species. From the establishment of China’s first giant panda reserves in 1963, 67 nature reserves (NRs) were established by 2015, covering approximately 54% of the giant panda’s habitat and protecting approximately 67% of the wild giant panda population [4]. Meanwhile, the Chinese government announced the official establishment of the Giant Panda National Park (GPNP) at the COP 15 meeting in 2021, which spans three provinces, Sichuan, Shaanxi, and Gansu, and covers a total area of 27,134 km^2^, with the aim of protecting the habitat of the giant panda and its sympatric species [5], marking a new phase in panda conservation. Although giant pandas have been downlisted from Endangered to Vulnerable in IUCN red lists, the Chinese government’s protection of pandas has increased rather than decreased [6], certainly setting an example for most of the world’s protected species.

**Figure 1:**
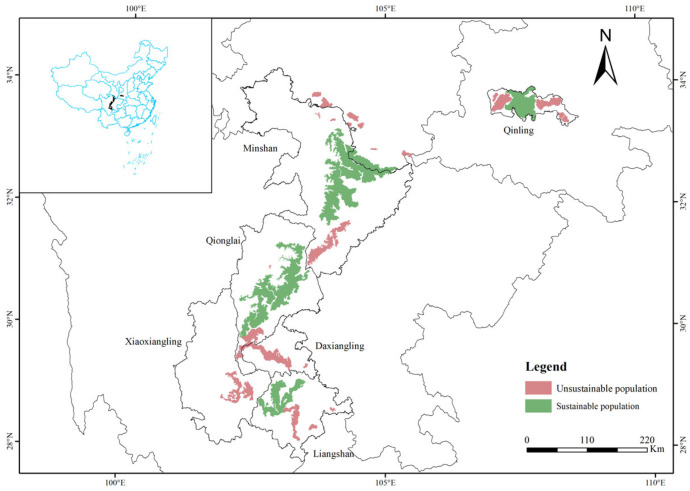
33 isolated populations of giant pandas in the six mountains.

According to The Fourth National Survey Report on the Giant Panda, there are 1864 pandas in the wild across China, separated into 33 local populations (Figure 1). Based on minimum area requirement (MAR) and minimal viable population (MVP), some of these populations are too small to be self-sustaining and, therefore, are at high risk of extinction [4]. Sustainable populations are those that meet the two conditions of panda population reaching its MVP and a habitat area greater than 114.7 km^2^ [7], of which there are 8 in total; unsustainable populations mean populations that do not meet both of these conditions, of which there are 25 in total. Clearly, the Chinese government is aware that the conservation of giant pandas is still a serious challenge. In parallel to building a more comprehensive conservation system for wild giant panda populations and their habitats, many active human interventions have been undertaken, including conservation translocation [8], damaged habitat restoration [9], and ecological corridor construction [10]. Of particular interest is conservation translocation, which refers to the intentional movement of organisms from one place to another.

There are many types of conservation translocations, including introduction, reintroduction, and reinforcement. Conservation translocation projects of charismatic species include the reintroduction of the California condor (*Gymnogyps californianus*) [11] and Lord Howe Island woodhen (*Hypotaenidia sylvestris*) [12]. However, available data for wildlife reintroductions suggest that the majority of programs suffer from weak planning, often insufficient monitoring, habitat-related issues, and failure to establish viable populations [13]. These drawbacks have led to the development of ‘reintroduction biology’ to bridge the research–management gap. Successful translocations should be guided by explicit theoretical frameworks based on clearly defined objectives and rigorous scientific studies [14].

**Figure 2:**
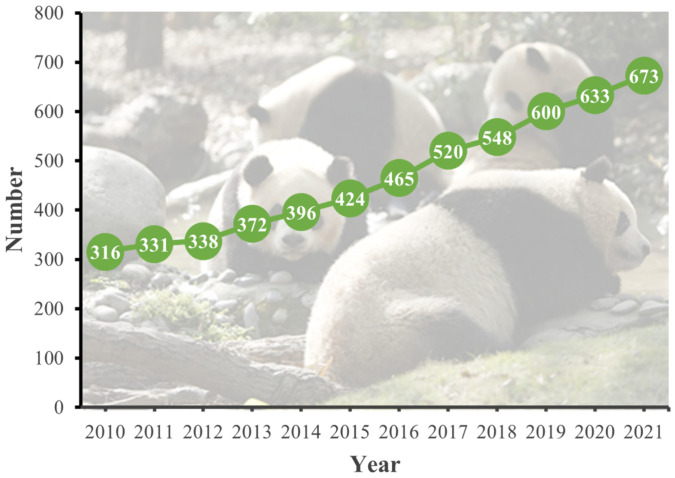
The population of captive giant pandas worldwide from 2010 to 2021.

Benefitting from the resolution of the so-called ‘trilemma’ of captive breeding, that is, difficult to rut, difficult to conceive, and difficult to raise young and survive, the captive-bred giant panda population has grown rapidly over the last 20 years, reaching 673 individuals by 2021 (Figure 2), laying the foundation for conservation translocation of captive-bred giant pandas into the wild. In fact, an international symposium on the feasibility of captive-bred giant panda reinforcement was held in Wolong in 1997; however, experts concluded that the conditions for releasing giant pandas into the wild were not yet met. In 2006, Zhang et al. (2006) concluded that the conditions for the release of captive giant pandas into the wild in China were primarily met, and the time was ripe for release [15]. However, the actual release of giant pandas in China began in 2005 with the rescue of “Shenglin 1” in the wild.

To date, a total of 15 releases have been recorded (Table 1), of which 3 were wild-born and 12 were captive-born; 3 individuals died (20%), and 12 survived (80%). The fact that all of the wild-born individuals survived and 3 of the captive-born individuals died seems to indicate that the former had a much higher chance of survival than the latter, although the sample size is too small to confirm this. The only wild-born individual with a reliable record is Luxin, who was released in 2009 at Liziping NR in Shimian. In the future, we should record reintroductions in the same way to increase the sample and knowledge base. Overall, conservation translocation of giant pandas in China is proceeding slowly. Before launching this complex and difficult procedure, managers and decision-makers need to clearly understand the rationale and technical challenges of conservation translocations, especially in the face of so many small populations with high extinction risk.

Fortunately, owing to the unique scientific and conservation value of the species, scientific research on giant pandas has garnered numerous studies, such as studies on habitat quality [16] and population dynamics [17] of giant pandas, covering both field and captive populations and different levels, from individual to landscape (Table 2). With the support Iof many scientific papers, the conservation biology of giant pandas can be said to have become a model case-study in the field of wildlife research. This article will introduce the selection of release sites and individuals for release, as well as pre-release and post-release work to explore the science underlying giant panda conservation translocations.

## 2. Selecting Suitable Release Sites

When identifying suitable sites for translocation, it is apparent that ‘species should never be released blindly without extensive assessment of habitat quality’ [18]. It is extremely important to select a suitable habitat for the released species in the process of conservation translocation [19]. Today, wild giant pandas are fragmented across six mountain systems in southwest China: the Qinling, Minshan, Qionglai, Daxiangling, Xiaoxiangling, and Liangshan mountains, and are separated into 33 small populations. To release captive giant pandas into the wild, we first need to understand the habitat requirements of giant pandas. According to existing studies, giant pandas usually choose old growth or secondary forests with medium to high densities of bamboo, medium altitudes and gentle slopes, and usually choose to avoid human disturbance and livestock [20,21,22,23]. In addition, forest age, topography, and the presence of bamboo were key predictor variables determining habitat selection by giant pandas at different scales [24,25].

Monitoring the released captive-bred giant panda Zhangxiang, Lei et al. (2015) found differences in dietary composition and microhabitat usage between Zhangxiang and local wild pandas. The percentage of bitten-not-broken bamboo was also larger from Zhangxiang than for wild pandas [26]. Thus, the ecological conditions of the release enclosure should be as similar as possible to the habitat of the population to be joined by the released individuals, otherwise the released individuals will face a process of behavioural adjustment and adaptation while integrating into the population. This is, in fact, a soft release process to maximise the chances of survival of the released individuals in the wild.

In addition to the above habitat requirements, these two other factors need to be a focused: MAR and MVP. MAR for a population is the area required for the long-term survival of that population. Qing et al. (2016) concluded that the MAR of giant panda populations is approximately 114.7 km^2^ [7]. Therefore, we need to upgrade the area of each habitat to at least this level to ensure that release sites are sufficient to sustain the growth of reintroduced populations in the long term [27]. A piece of counterevidence is provided by Xiangxiang; although it was released into an area with a favorable habitat, there was already a stable population of wild pandas. Xiangxiang may have died from competition with other wild individuals for territory and resources. Therefore, releasing pandas into an area which already has a dense existing population of pandas will be riskier because of competition and territoriality. Areas where wild populations have been extirpated or depleted should be given priority for receiving translocated individuals, but the reason for depletion must first be controlled before release.

Small populations are susceptible to large fluctuations caused by various levels of stochastic processes (e.g., genetics, environment, disasters, population statistics randomness) and are thus at risk of small population extinction [28]. Among the genetic problems for populations are inbreeding depression and loss of genetic diversity from genetic drift. Loss of genetic diversity can in turn be expected to cause a decreased ability in the population to adapt to environmental change and to survive outbreaks of disease, which is a huge blow to maintaining the dynamic balance of populations. Therefore, the number of pandas in each small population should reach a minimum value to cope with possible negative impacts, that is, the need to reach MVP. Further research is still needed on the calculation of MVP for wild giant panda populations. According to the Fourth National Survey Report on the Giant Panda, only 8 of the 33 populations meet the sustainability criteria; the remaining 25 populations are located in habitats where the risk of extinction can be reduced by releasing captive individuals or creating ecological corridors (Figure 1). The status of 25 local populations at risk of extinction and the recommended measures to prevent their extinction are as follows (Table 3). These measures are implemented on the basis that, as far as possible, the wild giant panda populations meet the MVP while their habitats meet the MAR.

## 3. Selecting Suitable Release Candidacy

### 3.1. Individual Source

Captive-bred animals fare relatively poorly in reintroduction programs [29]. They potentially have poor health and abnormal behaviour due to captivity [30] or a lack of important capacities, such as avoiding predators, foraging, and mating. A complete repertoire of survival-critical behaviours of free-ranging wild giant pandas should be assembled. Another avenue of interest is whether it is possible to translocate more wild individuals. The purpose of translocation is not to off-load captive animals or to justify captive breeding expenditures. Giant pandas are translocated to promote conservation. It may even be more conservation-friendly to move wild-caught animals from populations with a surplus of juveniles to populations below MVP, rather than reintroducing captive-bred animals. However, assessing the reasonableness of this idea requires more experimental studies.

### 3.2. Body Condition

The first thing to consider is the health of the release candidate. Pre-translocation parasite screening of wild populations and risk assessments are indispensable. For captive populations, selection for host tolerance can enhance the success of reintroduction or translocation [31]. The giant panda is known to be susceptible to natural infection with canine distemper virus (CDV). Bronson et al. (2007) recommend that giant pandas be vaccinated annually using the canarypox-vectored recombinant distemper vaccine [32]. Geng et al. (2020) found that giant panda cubs had the strongest immune response after the second vaccination [33]. Recently, Dai et al. (2021) also identified the complete genome sequence of a novel circovirus, giant panda-associated circovirus, in giant panda blood, but its pathogenesis in giant pandas needs to be further explored [34]. It is therefore essential that captive-bred individuals are thoroughly examined prior to release to select individuals more suitable for release, and be properly vaccinated to reduce the chance of infection by the virus.

### 3.3. Parasites and Viruses

Parasitic infection is another noteworthy issue for candidates. *Baylisascaris schroederi* is the most common parasite in wild and captive giant pandas, and the visceral larval migrants caused by *B. schroederi* infection are identified as the most significant threat to the survival of the giant panda, with the probability of death from this disease in the wild increasing significantly between 1971 and 2005 [35]. Zhu et al. (2020) used seven polymorphic functional Major Histocompatibility Complex (MHC) genes and found that heterozygotes and certain MHC variants were less likely to be infected with *B. schroederi*. When selecting individuals for release, this method can be used to select pandas that are less susceptible to disease, supplemented by the previous selection criteria, thus improving the chances of survival of released individuals in the wild [36]. Xinyuan, a two-year-old female who died from respiratory and renal failure before her release to Liziping NR, reminded us of the need for increased surveillance and research on the infectious diseases of giant pandas and the development of suitable vaccines [37]. In addition, there is always a serious risk that translocated animals will carry novel diseases into the wild and infect healthy populations [38]. This is one of the main risks that must be considered before translocation is carried out.

### 3.4. Sex and Age

Next, the sex and age of the release candidacy need to be considered. One genetic study suggest that female-biased dispersal occurs in giant pandas, most likely due to competition for birth dens among females, inbreeding avoidance, and enhanced inclusive fitness among related males [39]. This finding is supported by direct observation of a GPS-collared subadult female [40]. Moderate post-release dispersal is necessary for released individuals to survive in a changing environment and to promote gene flow among metapopulations [41]. This observation seems to suggest that the effect of releasing females is better than the effect of releasing males. Indeed, female pandas will directly take part in production and nurturing offspring. More females released can be expected to make a greater contribution to population recovery [42].

However, we should also consider the community structure of the target population for release, and that if there is a significant female bias in the community, then releasing males will be necessary. In theory, a single male panda could mate with several females and easily spread his genes widely among the target population. Therefore, mixed-sex combinations with female bias have better results than female-only or male-only combinations [8].

In addition, we should release subadult individuals at 3 to 4 years of age to keep the age structure of the target population for release growing and capable of normal reproductive activity. This is because they have, firstly, the basic ability to survive; secondly, sufficient time to establish home ranges, integrate into the community, and engage in learning about reproductive behaviour; and thirdly, the greatest contribution to population reinforcement [17].

### 3.5. Behaviour

Next, we also need to consider the behavioural factors of the release candidants. For behaviour, we focus on chemical communication behaviour and antipredatory behaviour. Chemical communication is important in pandas and there are multiple ways of marking, this includes anogenital gland secretion (AGS), urine, etc. The marks left behind contain information on individual status, sex, age, oestrus status, etc. [43,44]. Zhou et al. (2019) demonstrated that there is a difference in the chemical composition of AGS between captive and wild giant pandas and that this difference is most likely one of the important reasons for the low natural reproductive ability of captive giant pandas [45]. In this regard, we suggest that wild giant panda feces and marks from the release site can be collected for the release individuals to acclimatize to in advance.

Natural predators of giant pandas are mainly medium and large carnivores living in the same area, such as leopards (*Panthera pardus*), dholes (*Cuon alpinus*) and wolves (*Canis lupus*). Although Li et al. (2020) showed that the number of large carnivores in giant panda habitats is decreasing [46], mortality due to predation is a major cause of failure, and captive-bred giant pandas are isolated from predators throughout their lifetime and may no longer express antipredator behaviour [47]. Even though there is likely an innate component to predator recognition in captive giant pandas based on experiments using predator urine, antipredator training in prerelease preparation procedures is indispensable [48]. van Heezik et al. (1999) demonstrated that using a model predator was not an effective conditioning stimulus to houbara bustards (*Chlamydotis* [*undulata*] *macqueenii*), but pre-release training with a live predator significantly improved post-release survival [49]. Therefore, future research could explore whether live predators should be included in the pre-release training of giant pandas and whether they are more effective than model predators.

### 3.6. Genetic Consideration

Last but not least, for potential reintroduction, the maintenance of genetic diversity of captive-bred species through pedigree management has been increasingly addressed [50], but improving the exchange of genetic materials among institutions will be necessary for captive giant pandas because of lower levels of allelic diversity and heterozygosity in captive-bred populations compared to isolated wild populations [51]. For wild, isolated, and smaller populations of giant pandas with low genetic diversity which are facing a high level of inbreeding, genetic rescue can prevent the negative consequences of disrupted gene flow and isolation by increasing population size at population establishment or by gene flow as the population expands and connects with neighbouring populations [52,53]. For example, exchange between populations of Chengdu and Wanglang should be encouraged because of similar wild founder sources [54]. Whole genome sequencing of 34 wild giant pandas led to the classification of pandas into three distinct populations, Qinling (QIN), Minshan (MIN), and Qionglai–Daxiangling–Xiaoxiangling–Liangshan (QXL) [55], suggesting that different feasible schemes of recovery programs against three management units should be brought forwards. Individuals from different populations should not be cross-released to avoid the potential risk of outbreeding depression [54].

## 4. Pre-Release Training of Candidate

There are usually two ways of releasing captive animals, namely hard releasing and soft releasing [56]. At present, conservation translocations of giant pandas usually adopt soft release, which consists of these four main steps: prerelease training, acclimatization in enclosures, release, and post-release monitoring. Among these steps for release, pre-release training is widely considered to be the key to successful soft release [57]. Zhang et al. (2017) found that the inclusion of manual intervention in pr-release training caused differences in the behavioural development of released individuals compared to those individuals without manual intervention, things like habitat selection and home range [58]. To increase the success rate of soft release, we should minimize manual intervention in pre-release training.

In a study of the released individual Zhangxiang, Lei et al. (2015) found that there were significant differences in food habits between Zhangxiang and wild giant pandas during reintroduction training; it was less capable of handling bamboo than wild individuals, suggesting that Zhangxiang had to readapt to a new environment after release into the wild even after pre-release training, which inevitably reduced its chance of survival [26]. In view of this consideration, in the future, when building wild acclimatization pens, management should try to ensure that the site is similar to the wild environment that the released pandas will enter. In the end, it is important for captive pandas preparing for release to form as little dependence on people as possible [58]. For example, the recently popular ‘panda costume’, in which giant panda keepers disguise themselves as pandas to get closer to captive individuals, seems to be useful in preventing the imprinting of humans on panda cubs.

## 5. Post-Release Monitoring of Released Individuals

Post-release monitoring of released individuals is critical to ensure adequate population establishment, growth, and viability [14], and these metrics are commonly used to assess the success of translocation [59]. Monitoring of individual giant pandas released into the wild has commonly been carried out, especially in the Liziping NR [42]. To date, many advanced technologies have been used for post-release monitoring, such as GPS collars, camera trapping technology, genetic monitoring, and automatic giant panda identification systems (a new Feature-Fusion Network with Patch Detector) [8,60,61]. Post-release monitoring actually includes these two aspects: monitoring of released individuals and monitoring of population dynamics, but most of the current monitoring is directed at the former, and research on the latter is lacking. The purpose of releasing captive giant pandas in the wild is to form self-sustaining populations to avoid the extinction of small populations [14,38], so monitoring population dynamics is essential, and research in this area should be strengthened in the future. Bubac et al. (2019) also found that most translocation studies were conducted for only 1–4 years of post-release monitoring, with the highest proportion of failures occurring in the first 4 years [13]. Based on this finding, and in conjunction with existing research on the release of giant pandas, we should monitor captive giant pandas for a longer period of time after their release into the wild.

## 6. Future Directions

Although we are currently able to select suitable release sites for individuals about to be released, one more thing we should do in the future is to determine the factors causing the depletion of the original population at the release site. Habitat degradation and fragmentation are well known to threaten the sustainable survival of giant pandas [62], but we should determine which specific factors are causing pandas to become unsuitable for distribution in this area, such as changes in food (bamboo), breeding dens, climate, and forest cover [10,63,64,65]. Only after this determination can we take measures to make the release site suitable for releasing individuals to form self-sustaining populations. In addition, the multiscale Maxent approach can help conservationists model the habitat suitability of giant pandas, which can guide the selection of release sites [66]. Surveys between releases are therefore necessary, and in some areas, some active human interventions may have to be undertaken, such as habitat expansion, construction of giant panda breeding dens, improving bamboo forest growth, etc.

Reintroduction by releasing captive-bred individuals to recover wild giant panda populations within their historical distribution range is the most common approach [15]. Applications of other conservation translocations of giant pandas need to be explored by reliable model predictions, even though it is extremely challenging. At present, before the conservation translocations of giant pandas, we should first focus on restoring suitable habitats and building ecological corridors so that existing giant panda populations have sufficient resources to sustain themselves, which is the primary way to protect wild giant panda populations.

Morris et al. (2021) summarized what is known about conservation translocation projects and concluded that factors such as the number of individuals released, the geographical location of the release, and the characteristics of the released species can affect the success of conservation translocations of terrestrial vertebrates [67]. Facing the complex ecological requirements (habitat quality, food resources, predator control, breeding dens, and climate change) of giant pandas, the best methods for selecting suitable release sites are often uncertain. Adaptive management (AM) can be helpful in the face of such uncertainty by balancing the benefits of improved information against the goals of management [68]. We see great potential for increasing applications of AM to improve the success of giant panda conservation translocation programs. AM can recognise and combat uncertainty and help us make better decisions [69].

## 7. Conclusions

Overall, the conservation of giant pandas is changing from passive in situ conservation to active conservation with human intervention, and is moving from crude to more precise approaches. In the foreseeable future, scientific research will become increasingly prominent in the development of giant panda conservation. As one of the key instruments for the maintenance of wild giant panda populations, conservation translocation should be actively promoted and continuously improved. As a core issue, improving the survival rate of released individuals in the wild is crucial. This study provides a theoretical basis for improving the survival of captive giant panda individuals in the wild by reviewing aspects such as the selection of individuals for release and release sites. However, before the conservation translocations of pandas, we should first focus on restoring suitable habitats and building ecological corridors so that existing populations have sufficient resources to sustain themselves, which is the primary way to protect wild populations. As a model species, the giant panda also provides a reference for the global conservation of rare and endangered species in conservation translocation, and we are actively providing Chinese wisdom towards global biodiversity conservation.

## Figures and Tables

**Table 1:** The release of giant pandas in China to date.

Name	Gender	Source	Release Year	Release Site	Survival Status
Shenglin 1	Female	Wild-born	2005	Longxi-hongkou NR	Still alive
Xiangxiang	Male	Captive-born	2006	Wolong NR	Died (fights and injuries)
Luxin	Female	Wild-born	2009	Liziping NR	Still alive (produced offspring)
Taotao	Male	Captive-born	2012	Liziping NR	Still alive
Zhangxiang	Female	Captive-born	2013	Liziping NR	Still alive
Xuexue	Female	Captive-born	2014	Liziping NR	Died (conditional pathogenic infections)
Huajiao	Female	Captive-born	2015	Liziping NR	Still alive
Hesheng	Male	Captive-born	2016	Liziping NR	Died (attacked by unknown animal)
Huayan	Female	Captive-born	2016	Liziping NR	Still alive
Zhangmeng	Female	Captive-born	2016	Liziping NR	Still alive
Yingxue	Female	Captive-born	2017	Liziping NR	Still alive
Baxi	Male	Captive-born	2017	Liziping NR	Still alive
Qinxin	Female	Captive-born	2018	Longxi-hongkou NR	Still alive
Xiaohetao	Female	Captive-born	2018	Longxi-hongkou NR	Still alive
Tangtang	Female	Wild-born	2021	Foping NR	Still alive

Note: In brackets are the cause of death of the dead individuals and the current status of the surviving individuals. Survival status of giant pandas until October 2023.

**Table 2:** Research topics on giant pandas and published papers examined from 2000 to 2022.

Research Topic	Number of Papers from Different Sources		
	Science Direct	Web of Science	CNKI
Wild giant panda	1471	282	653
Captive giant panda	551	234	637
Giant panda	3330	1398	9462
Giant panda population	2036	347	1144
Giant panda landscape	625	98	46

CNKI: China National Knowledge Infrastructure.

**Table 3:** Local populations at risk of extinction and their mitigation measures.

Mountain Ranges	Populations Name	Number of Extant Giant Pandas	Habitat Area (km^2^)	Suggested Mitigation Measures
Qinling	Qinling A	7	211.41	1
	Qinling B	20	594.22	1
	Qinling D	36	641.01	1
	Qinling E	3	90.47	1 + 2
	Qinling F	4	128.45	1 + 2
Minshan	Minshan A	4	135.93	1 + 2
	Minshan B	9	238.81	1
	Minshan C	3	204.89	1
	Minshan D	1	56.00	2
	Minshan E	2	93.39	2
	Minshan F	2	36.07	2
	Minshan H	2	34.06	2
	Minshan I	1	33.69	2
	Minshan L	35	1363.99	1
Qionglai	Qionglai D	29	800.44	1
	Qionglai E	1	17.27	2
Daxiangling	Daxiangling A	4	205.75	1
	Daxiangling B	32	979.16	1
	Daxiangling C	2	43.78	2
Xiaoxiangling	Xiaoxiangling A	21	442.31	1
	Xiaoxiangling B	9	751.33	1
Liangshan	Liangshan B	22	528.41	1
	Liangshan C	3	173.60	1
	Liangshan D	4	107.59	1 + 2
	Liangshan E	3	48.12	2

Note: 1 represents the release of captive-bred individuals; 2 represents the establishment of ecological corridors. All data and populations name were obtained from the Fourth National Survey Report on the Giant Panda.

## Data Availability

Not applicable.
